# Electrospinning of n-hemin/PAN Nanocomposite Membranes and Its Photo-Enhanced Enzyme-like Catalysis

**DOI:** 10.3390/polym14235135

**Published:** 2022-11-25

**Authors:** Xu Han, Yun Tao, Chao Xu, Yicong Deng, Zisen Meng, Zhenhao Dou, Peng Wang, Quan Feng

**Affiliations:** 1 School of Textile and Garment, Anhui Polytechnic University, Wuhu 241000, China; 2 Advanced Fiber Materials Engineering Research Center of Anhui Province, Anhui Polytechnic University, Wuhu 241000, China

**Keywords:** polyacrylonitrile, hemin, photocatalysis, enzyme-like catalysis, electrospinning

## Abstract

Hemin possesses great potential in eliminating organic pollutants due to its mild reaction condition, light-harvesting efficiency, and environmental friendliness. However, it has drawbacks such as being easy to aggregate and hard to recycle, and poor stability should be improved in practical application. Herein, the subject developed an electrospinning approach to enable the hemin particulates to be immobilized onto polyacrylonitrile (PAN) nanofibers stably. Hydrogen peroxide (H_2_O_2_) was adopted as an oxidant in the system to simulate the enzymatic catalysis of hemin in an organism. Scanning electron microscopy (SEM), Fourier transform infrared spectroscopy (FTIR), X-ray photoelectron spectroscopy (XPS), UV-Vis diffuse reflection spectroscopy (DRS), and electron spin resonance spectroscopy (ESR) analysis was employed to discuss the morphology, structure, and mechanism of the prepared n-hemin/PAN nanocomposite membranes, and 0.02 mmol L^−1^ of the rhodamine B (RhB) removal activity in different conditions was also verified with these membranes. The kinetic studies showed that n-hemin/PAN nanocomposite membranes maintained excellent properties both in adsorption and degradation. Around 42% RhB could be adsorbed in the dark, while 91% RhB decolorized under xenon lamp irradiation in 110 min, suggesting the catalytic performance of n-hemin/PAN was greatly driven by light irradiation. Differing from the axial coordinated hemin complexes, n-hemin/PAN would catalyze hydrogen peroxide into •OH radicals rather than •OOH and high-valent metal-oxo species. This work provides an effective way to support hemin as nanocomposite membranes, in which the molecular interaction between polymer and hemin made their light adsorption an obvious red shift.

## 1. Introduction

Industries such as food, textile, and pharmaceuticals, etc., discharge a variety of organic effluents as wastewater into the environment, with features such as high colority, broad pH range fluctuations, and complicated composition [1,2]. Photocatalysis and photo-Fenton reactions convert absorbed light energy to chemical energy so as to produce active radicals, such as •OH and •OOH, oxidizing organics into harmless smaller molecules [3,4]. However, these photo-enhanced processes should still confront some deficiencies. For instance, homogeneous catalyst particles suffer from recycling issues, while the metal-based catalysts are restricted to pH variation. Oxidases and oxygenases exist in living organisms, and their catalytic oxidation efficiency is superior compared to the metal catalysts [5]. Studies have verified that oxidases and oxygenases are composed of a transition metal atom or metal cluster, with structures such as protein or a DNA skeleton, in which the special structure between metal atoms and amino acid residues determine their excellent catalytic activity [6,7]. Hence, the rational utilization of oxidases and oxygenases by appropriate methods and the in-depth study of their catalytic mechanism will have important guiding significance for the improvement of traditional catalysts and the performance development of new photo-enhanced enzyme catalysts.

Hemin, as a biological enzyme extracted from living organisms, can effectively catalyze the oxygen into oxidizing species, i.e., •OH and high-valent metal-oxo species [8,9,10,11]. Due to its high activity and selectivity in producing reactive species, numerous studies [12,13,14,15] have been carried out by developing hemin as a biomimetic catalyst or a visible light-enhanced catalyst, to substitute the traditional biodegradation method in eliminating organic wastewater. Zhu et al. [16] grafted hemin on the channeled mesoporous silica of SBA-15 to prepare an enzyme mimic catalyst, which showed effective activity in degrading dyes in the presence of H_2_O_2_. Moreover, the composite displayed high loading content and sustainable releasing behavior of the anticancer drug doxorubicin hydrochloride, resulting in promising biomedical application. Xu and co-workers [17] coupled hemin to the carbon skeleton via a covalent bond to synthesize a kind of functionalized carbon nanobranch (CN-hemin), which showed intrinsic peroxidase-like activity and satisfactory photocatalytic performance. Importantly, the hybrids showed effective performance for synergistic dye degradation and antibacterial therapy toward *S. aureus* and methicillin-resistant *S. aureus*. Hou et al. [18] prepared multiwall carbon nanotube decorated hemin/Mn-MOF by immobilizing hemin and a multiwall carbon nano tube on the pre-prepared Mn-MOF successively. The Fe atoms of hemin interacted with Mn to accelerate the redox reaction of the catalysis process and, as a result, CNT-hemin/Mn-MOFs showed an excellent catalytic effect in degrading BPA within 30 min.

The catalytic principle of hemin is similar to that of an iron-porphyrin complex. However, hemin particulates are easy to aggregate together as bulk powders under strong light irradiation, and warm water stirring, etc., which would significantly decrease their catalytic performance [19,20]. Additionally, hemin is easily destructed by radicals generated by itself, especially the dissociative •OH radicals. Thus, one effective solution to overcome the drawbacks is to immobilize hemin particulates onto a certain support, achieving heterogeneous catalysis. Most of the reported studies load hemin onto support such as carbon fiber [21,22], chitosan [23,24], or montmorillonite [25], in which hemin also suffers from a catalyst deactivation problem due to the unstable linkage between the catalyst and carrier.

Electrospinning is a convenient and efficient technique to immobilize a catalyst onto polymer fiber [26,27,28,29,30], and the supported particles would be adhesively fixed onto the surface of the nanofibers during the fibers’ formation process, or bonded onto active groups of polymers by coordination [31,32], a hydrogen bridge [33], a covalent bond [34,35], etc. In addition, polyacrylonitrile has excellent mechanical properties, chemical tolerance, and high affinity to organic pollutants, especially its light resistance ability, which makes it a great potential polymer support for a photo-enhanced catalyst [36,37]. In our previous study, iron phthalocyanine (FePc) particles were immobilized onto the surface of PAN yarns through the fifth ligand coordination between hydroxyl groups from modified PAN and Fe atoms from FePc, which showed stable and efficient catalysis in degrading dyes [38,39,40]. However, the active performance of FePc-PAN was hard to improve, which is mainly restricted by the size effect of PAN yarns.

Herein, the current work aimed to prepare a kind of novel catalyst, n-hemin/PAN, to degrade environmental pollutants by one-step preparation, immobilizing a hemin catalyst onto PAN nanofibers through electrospinning. RhB was used as a target compound to evaluate the photocatalytic performance of n-hemin/PAN. The oxygen of the oxidants was replaced with hydrogen peroxide to mimic the enzyme-like catalysis and accelerate its degradation efficiency. To further induce its catalytic activity, visible light was introduced in the pollutants-eliminating process to achieve an ideal photo-enhanced enzyme-like catalytic system. Above all, in the catalytic reaction system, H_2_O_2_ would be catalyzed by n-hemin/PAN in the presence of visible light irradiation to produce oxidative radicals, and thus continuously degrade RhB into small molecules. SEM, FTIR, XPS, DRS, and ESR techniques were also conducted in this subject to study the morphology, structure, and mechanism of the prepared catalyst samples. The adsorption and catalytic activity of the hemin-based catalysts were also discussed under dark and irradiation states, to evaluate their mechanisms in removing organic pollutants. The investigation is proposed to signify an inspiration to conduct a pathway for improving the enzyme-like catalysis of hemin by supporting it onto polymers to be an even fibrous catalyst, the interaction between molecules might also improve their light absorbance. Such results are signified as a driving force for full-scale real industrial applications.

## 2. Materials and Methods

### 2.1. Materials

Polyacrylonitrile (copolymer, M.W. 80,000) was purchased from HaoRui Chemical (Shanghai) Co., Ltd., Shanghai, China. Hemin (≥99.5%), N,N-dimethylformamide (≥99.5%), ethanol (≥99.5%), hydrogen peroxide (≥30%), rhodamine B (≥99.9%), dimethyl sulfoxide (≥99.9%), 5, 5-Dietyl-1-Pyrroline-N-oxide (DMPO, ≥97.0%) were purchased from Shanghai aladdin biochemical technology Co., Ltd., Shanghai, China.

### 2.2. Fabrication of n-hemin/PAN

**Figure 1:**
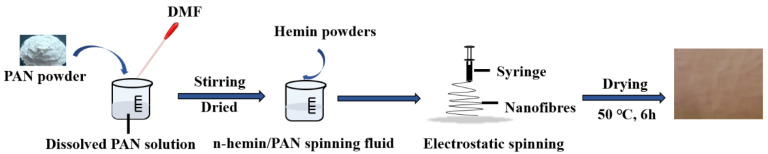
The preparation diagram of n-hemin/PAN.

To remove the impurities in the polyacrylonitrile powder, it was immersed into a beaker which had been filled with distilled water and ethanol with a volume ratio of 1:1. After stirring at room temperature for 30 min, it was removed and washed repeatedly with distilled water, then dried at 50 °C for 24 h. Subsequently, the pretreated PAN fibers were dissolved in N,N-dimethylformamide solvent to prepare the PAN spinning solution with a mass fraction of 10%. On this basis, hemin powders with concentrations of 2 g L^−1^, 20 g L^−1^ and 40 g L^−1^ were added to the PAN spinning solution and totally dissolved by stirring. An electrospinning process was conducted to prepare the n-hemin/PAN nanocomposite membranes, and the electrospinning conditions were as follows: the spinning voltage was 15 kV, the extrusion rate was 0.8 mL/h, the distance between receiver and needle was 25 cm, the environmental temperature was 25 ± 2 °C, and the humidity was around 60%. The residual solvent on the nanofibers was removed by an oven at 50 °C for 6 h. The fabrication of n-hemin/PAN is illustrated in Figure 1.

### 2.3. Characterization Techniques

The morphology of a series of n-hemin/PAN nanocomposite membranes was examined by an S4800 field emission scanning electron microscope (Hitachi, Ltd., Tokyo, Japan). The Fourier transform infrared spectra of the membranes were measured on a Nexus 670 Fourier transform spectrometer (Nicolet Inc., Glendale, WI, USA). The X-ray photoelectron spectroscopy was employed by using a PHI 5600 X-ray photoelectron spectrometer (Perkin Elmer Inc., Waltham, MA, USA). The diffuse reflectance UV-Vis absorption spectra in the range from 200 to 800 nm were investigated by using Varian Cary 500 UV-vis spectrophotometer (Thermo Fisher Scientific Inc., Waltham, MA, USA). The total organic carbon (TOC) throughout the catalytic process was analyzed by using Shimadzu TOC-L (Shimadzu, Ltd., Kyoto, Japan). The atomic absorption spectrum was conducted by using a Shimadzu AA-7000 (Shimadzu, Ltd., Kyoto, Japan) to detect the residual Fe ions in the dye solution.

### 2.4. Catalytic Performance Measurement

The catalytic performance of the samples was tested through a purchased photochemical reactor, which consisted mainly of a xenon lamp, relay, reactor, water bath, and water cooling system, etc. The temperature during the reaction process was controlled at 25 ± 1 °C. The light irradiation intensity was measured by an FZ-A radiometer (BNU Light and Electronic Instrumental Co., Beijing, China). The xenon lamp (500 W) in the purchased photochemical reactor was able to simulate the full spectrum of sunlight. Additionally, a cut-off filter was used between the membranes and the light source to ensure the samples were irradiated only by visible light (λ > 420 nm). RhB dyes with a concentration of 0.02 mmol L^−1^ were adopted as the simulated contaminant, and its decoloration test was performed by adding 0.1 g n-hemin/PAN membrane into 50 mL RhB solution without stirring. Samples were suspended in the solution. At given time intervals, a visible spectrophotometer was used to analyze the Abs values of the solution. The visible light intensity ranged from 1.83–11.46 mW/cm^2^ by adjusting the power of the lamp, while the H_2_O_2_ concentration in the solution was adjusted to 25, 35, and 45 mmol L^−1^, respectively. The experiments were repeated three times for each sample. After recording the absorbance of λ_max_ (550 nm) for a specific dye solution, the decoloration rate was calculated as: D% = (1 − C/C_0_) × 100%, where C and C_0_ corresponded to the residual and initial dye concentration, respectively.

## 3. Results and Discussion

### 3.1. Characterization

#### 3.1.1. SEM

**Figure 2:**
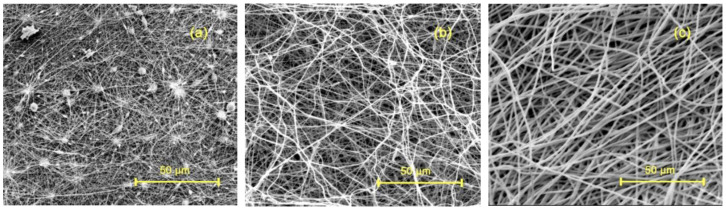
The effect of PAN mass fraction on the morphology of n-hemin/PAN: (**a**) 8%PAN, (**b**) 14% PAN, (**c**) 20%PAN.

To investigate the effect of the mass fraction on the morphology of n-hemin/PAN, PAN spinning solutions with mass fractions of 8%, 14%, and 20% were prepared, and the result is shown in Figure 1. It was clear to see that the n-hemin/PAN composite nanofiber membrane’s spinning property was improved with the rise of PAN solution concentration. When the mass fraction was 8% (Figure 2a), the viscosity of the spinning solution was relatively low, thus the volatilization of DMF solvent was incomplete. As a result, the surface morphology of the fiber membrane was irregular and numerous particles and spindle-like polymers were found between fibers, this was due to the incomplete solvent volatilization during the spinning solution’s spray process. These defects had an impact on the mechanical properties of the membrane. Figure 2b shows that the spindle-like beads disappeared when the mass fraction was 14%. With the appropriate viscosity, the morphology of the membrane became more curved and intertwined with each other. According to the calculation from Image J, the fiber’s average diameter of 14% PAN distributes around 250 nm. It can be seen from Figure 2c that when the mass fraction grows to 20%, the membrane shows smoother morphology, the fiber’s orientation is more uniform without obvious tangling, and the fiber’s diameter increases significantly to the micron level.

For the sake of investigating the effect of hemin content in n-hemin/PAN on its apparent morphology, hemin molecules with concentrations of 0, 2, 20, and 40 g L^−1^ were added to the PAN spinning solution, respectively. The mass fraction of PAN in these spinning solutions was controlled at 14%. The ferric ions content in the serial membranes was detected through phenanthroline photometry, and the result showed that n-hemin/PAN membranes with hemin concentrations of 2, 20, and 40 g L^−1^ in the electrospinning solution had an Fe ions content of 0.8, 14.3, 29.1 mg g^−1^, respectively.

**Figure 3:**
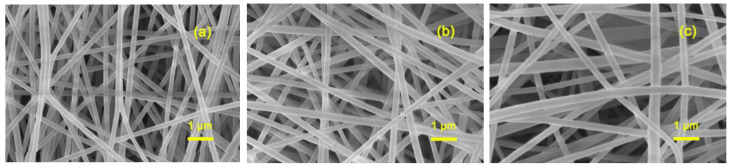
SEM micrographs of n-hemin/PAN with different hemin mass fractions: (**a**) 0.8 mg g^−1^; (**b**) 14.3 mg g^−1^, (**c**) 29.1 mg g^−1^.

In order to further analyze the microstructures of the n-hemin/PAN membranes, an SEM was carried out and the results are shown in Figure 3. As can be seen when C_Fe_ increased from 0.8 mg g^−1^ to 29.1 mg g^−1^, the composite membrane n-hemin/PAN still maintains an even and smooth appearance, which was barely affected by the loading account of hemin, suggesting PAN possesses good compatibility with hemin molecules. Based on calculations from Image J, the fiber diameters of n-hemin/PAN with a hemin concentration of 0.8, 14.3, and 29.1 mg g^−1^ were 190, 225, and 270 nm, respectively.

#### 3.1.2. FTIR

**Figure 4:**
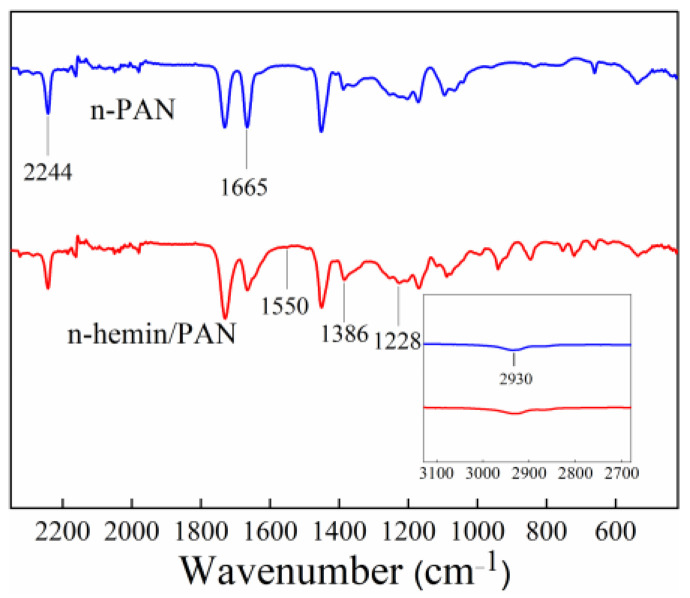
FTIR spectra of n-PAN and n-hemin/PAN.

For investigating the preparation and microstructure of the composite membranes, comparative FTIR analysis between n-PAN and n-hemin/PAN was carried out, and the result is illustrated in Figure 4. The n-PAN nanofiber membrane shows its characteristic absorption at 2244 cm^−1^, which corresponds to the stretching vibration of the -CN group. The peaks at 2930 and 1665 cm^−1^ were attributed to the stretching vibration of the C-H bond in saturated carbon and the C=O bond in the third monomer carboxylic acid of PAN [41], respectively. On the other hand, the n-hemin/PAN membrane shows obvious differences to the n-PAN in the wavenumber range of 600–1700 cm^−1^. For instance, the absorptive peak at 1386 cm^−1^ was assigned to the B_3u_ vibration of porphyrin [42], while the peaks at 1550 and 1228 cm^−1^ were ascribed to the stretching vibration C=C, C=N bonds in the ring and the in-plane vibration of C-H bonds [43], respectively. It was noteworthy that, in C=O bonds, peak intensity decreased, while the half-peak width increased significantly when hemin was composited in the polymer, manifesting that there may be molecular interaction between the unsaturated bonds of atoms in hemin and the C=O bonds in n-PAN. Due to the conjugation of the O atom with lone pair electrons, the electron cloud on C=O was shifted to the O atom, which leads to the equalization of the electron cloud density for the C=O double bond. As a result, the force constant of C=O bond decreased, and the characteristic absorption shifted to a lower wavelength.

#### 3.1.3. XPS

**Figure 5:**
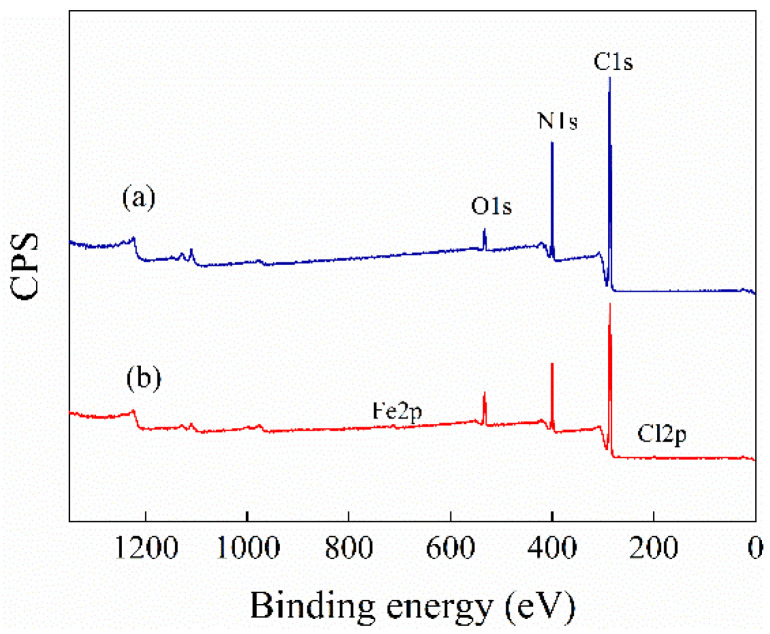
XPS survey spectra of n-PAN (**a**) and n-hemin/PAN (**b**).

**Figure 6:**
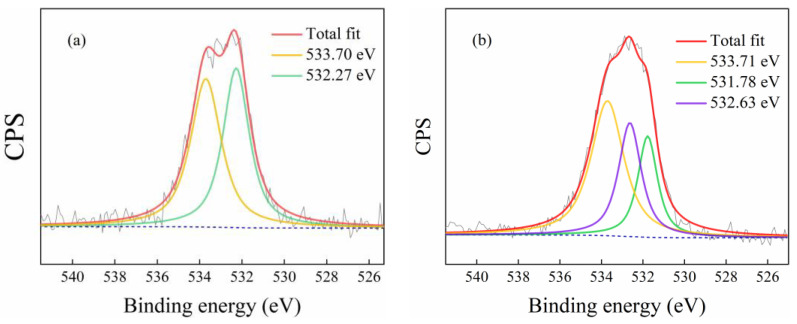
XPS O1s spectra of n-PAN (**a**) and n-hemin/PAN (**b**).

To further verify the interactions between n-PAN and n-hemin/PAN, XPS analysis on the surface of these samples was carried out, as illustrated in Figure 5 and Figure 6. Figure 5 is the full XPS spectrum for both samples, which showed the characteristic signal for O1s, N1s, and C1s on the binding energy of 400.0, 286.8, and 533.1 eV, respectively. After the composition between PAN and hemin in the nanofiber membranes, n-hemin/PAN showed new peaks at 199.8 eV and 712.34 eV, which was attributed to the XPS signals of Cl 2p and Fe 2p in hemin molecules, respectively, indicating hemin molecules were successfully loaded on the fiber surface.

Figure 6 illustrates the comparative study for n-PAN and n-hemin/PAN on O1s spectra, and the result was processed through XPSpeak 4.1. The O1s binding energy for n-PAN could be divided into two peaks at 533.70 eV, and 532.27 eV, respectively, which corresponded to oxygen atoms in C-O-Na and C=O groups. On the other hand, the O1s binding energy for n-hemin/PAN was composed by three peaks at 533.71 eV, 531.78 eV, and 532.63 eV, assigning to the oxygen atoms in the PAN polymer C-O-Na, C=O and the carboxyl oxygen atoms in hemin [40], respectively. It was found that the O1s binding energy for C=O in n-PAN decreased from 532.27 eV to 531.78 eV, suggesting the conjugated π bonds between carboxyl groups in PAN and unsaturated bond in hemin molecules may be formed. Carboxyl oxygen in PAN had a strong electron absorption ability which could attract the electrons in hemin molecules shifting to itself, and thus increases the electron cloud density around it, and decreases its binding energy. The result was consistent with the analysis results in FTIR.

#### 3.1.4. DRS

**Figure 7:**
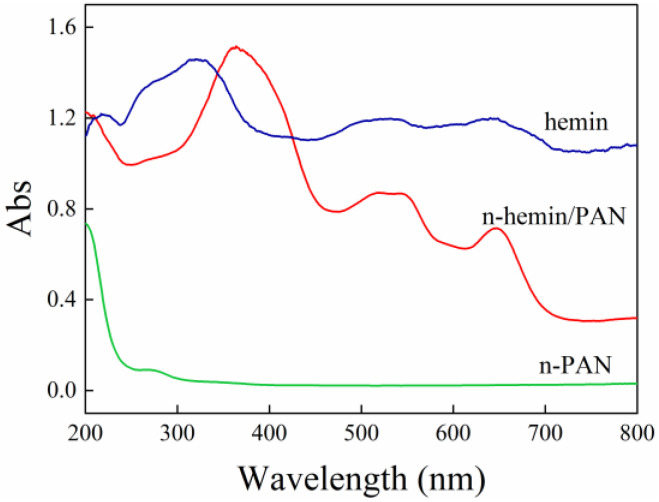
DRS spectra of n-PAN, hemin, n-hemin/PAN.

Chen’s study [44] confirmed hemin had excellent catalytic performance under the activation of ultraviolet and visible light. Hereby, the DRS spectra for hemin, n-PAN, and n-hemin/PAN were investigated, and the results are shown in Figure 7. The n-PAN membrane appears to have a strong absorption peak at 200 nm corresponding to the π-π* electronic transition of C≡N bond, and one peak corresponding to the n-π* transition of C=O around 270 nm shows relatively weak intensity since the n-π* transition is sterically resistant. In addition, the hemin powder shows excellent optical absorption performance in the range of 200–800 nm. The strong peak around 320 nm, and the relatively weak peak at 525 nm and 638 nm, is attributed to the B-band and Q-band absorption peak of hemin, respectively. Furthermore, when hemin interacted with PAN in the n-hemin/PAN membrane, the n-hemin/PAN inherited the optical performance of hemin in the range of UV and visible light, and showed strong absorption peaks at 360 nm, 530 nm, and 650 nm, in which 360 nm belonged to the B-band absorption, while 530 nm and 650 nm belonged to Q band absorption. It was found from comparing the study of n-PAN and hemin that the B-band absorption for hemin showed a surprisingly red shift in n-hemin/PAN, which would accelerate the photo-response ability for hemin significantly. The reason for this is due to the conjugated π bond interaction between polymers and hemin, as was discussed in the FTIR study. The DRS results also provide explanation for the high catalysis performance of n-hemin/PAN under visible light irradiation.

**Figure 8:**
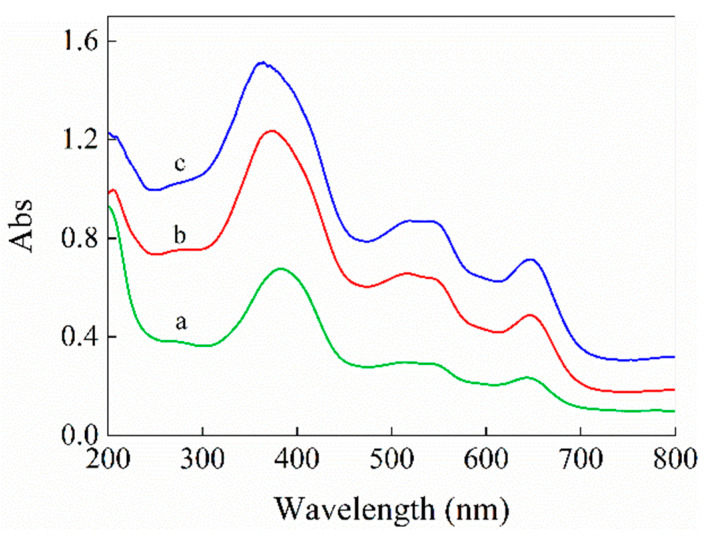
DRS spectra of n-hemin/PAN with different hemin loading capacities: (**a**) 0.8 mg g^−1^, (**b**) 14.3 mg g^−1^, (**c**) 29.1 mg g^−1^.

To further investigate the effect of the hemin loading capacity of n-hemin/PAN on its optical absorption property, comparative study to the DRS spectra of the n-hemin/PAN composite nanofiber membrane with different hemin account was carried out, as is shown in Figure 8. With an increase in the hemin account, absorption intensity of the n-hemin/PAN membrane enhanced significantly in the range of 200–800 nm. Moreover, the characteristic peak for B-band around 360 nm shows a blue shift with the increase of hemin loading capacity, which is due to the intrinsic absorption of hemin as the increase of its content.

### 3.2. Catalytic Properties of n-hemin/PAN

**Figure 9:**
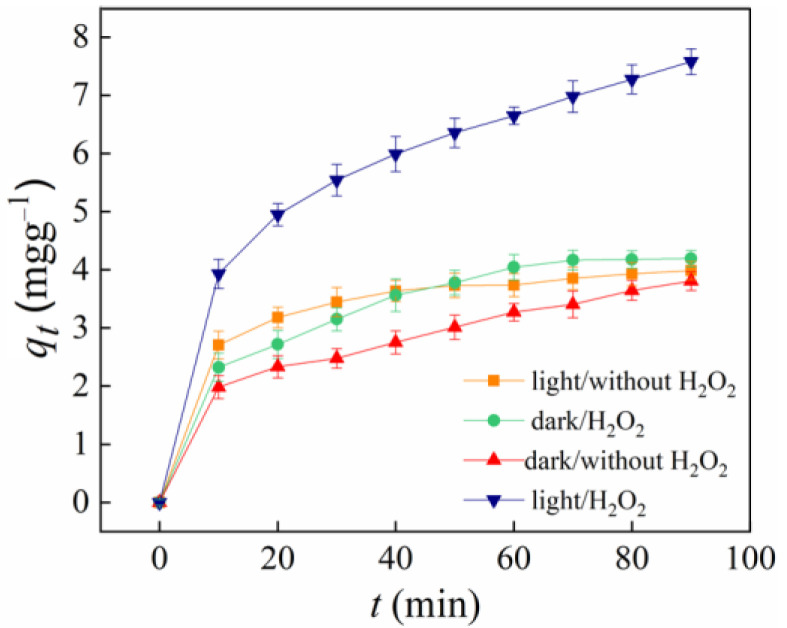
Adsorption/catalytic performance of n-hemin/PAN in different reaction conditions: dark/without H_2_O_2_, light/without H_2_O_2_, dark/H_2_O_2_, and light/H_2_O_2_.

According to the SEM characterization, the average diameter of an n-hemin/PAN fiber distributes from 190–270 nm, which is significantly smaller compared to the traditional fibrous textiles such as nonwovens or yarns. The nanoscale diameter of the membrane inevitably leads to the enlargement of the n-hemin/PAN specific surface area, which would significantly improve its adsorption performance. Thus, a 0.02 g membrane was adopted in 20 mL RhB solution with a concentration of 0.02 mmol L^−1^ to study the potential adsorption performance of n-hemin/PAN, adsorption processes in different conditions were discussed. As is shown in Figure 9, in order to distinguish the contribution of adsorption and catalysis for an n-hemin/PAN composite membrane, RhB solution was treated under a series of conditions: dark/without H_2_O_2_, light/without H_2_O_2_, dark/H_2_O_2_, and light/H_2_O_2_. The results showed that the RhB adsorption equilibrium using n-hemin/PAN was around 4 mg g^−1^, while the dye decolorization rate was about 42%, indicating that n-hemin/PAN had a significant adsorption effect on RhB dye. It is due to the strong attraction between the electronegative carboxyl groups in the third monomer of PAN and the electropositive charges from RhB dyes. Moreover, the comparative study between the curves of dark/H_2_O_2_ and dark/without H_2_O_2_ showed that n-hemin/PAN could hardly undergo catalytic performance in the dark state, suggesting the catalyst was mainly driven by light irradiation.

**Figure 10:**
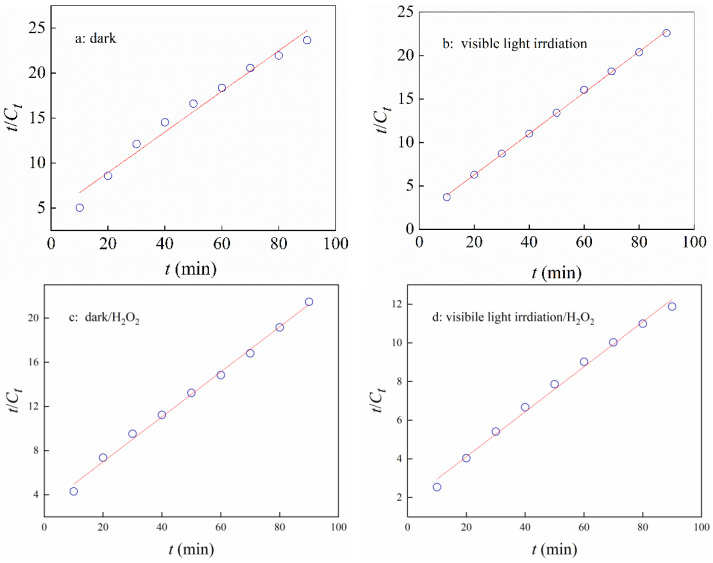
Pseudo-second-order equation plots of n-hemin/PAN: (**a**) dark without H_2_O_2_, (**b**) light irradiation without H_2_O_2_, (**c**) dark with H_2_O_2_, (**d**) light irradiation with H_2_O_2_.

To further verify the adsorption/catalytic performance of the n-hemin/PAN composite membrane under different conditions, a pseudo-second-order equation was used to fit the above data. Accordingly, the relationship between the adsorption rate of RhB on the n-hemin/PAN surface and the number of unoccupied adsorption vacancies on the fibers are as follows:

t/q_t_ = 1/kq_e_^2^ + t/q_e_ (1)

where, q_t_ and q_e_ are the total mass of RhB removed during the catalytic and adsorptive process by per mass of n-hemin/PAN, respectively, and k is the pseudo-second-order kinetic rate constant. Based on the data from Figure 9, time t was plotted with t/qt and the results were processed by linear fitting, then the intercept and slope was obtained as values k and q_e_, the results were shown in Figure 10 and Table 1.

The analytic data from above reveals that the correlation coefficients R^2^ obtained by pseudo-second-order equation were bigger than 0.99 under the conditions of a, b, and c, and the q_e_ values fitted from these three reactions were basically consistent with the experimental value, confirming that the adsorption process of the dyes follows the pseudo-second-order equation when light and H_2_O_2_ do not coexist simultaneously. In addition, the comparative study for the constant k derived from reaction a, b, and c showed that light may accelerate the adsorption rate of n-hemin/PAN where H_2_O_2_ barely affects the process. Notably, although the decoloration rate of hemin-PAN under the visible irradiation and H_2_O_2_ condition was significantly higher than the others, the constant k was an order of magnitude smaller, indicating the catalytic property of the membranes possesses a major role other than adsorption in the condition. The result was in accordance with the R^2^ value in reaction d (0.9731), which certified the decoloration mechanism of n-hemin/PAN under visible irradiation and H_2_O_2_ was a synergy reaction within the adsorption and catalysis.

**Figure 11:**
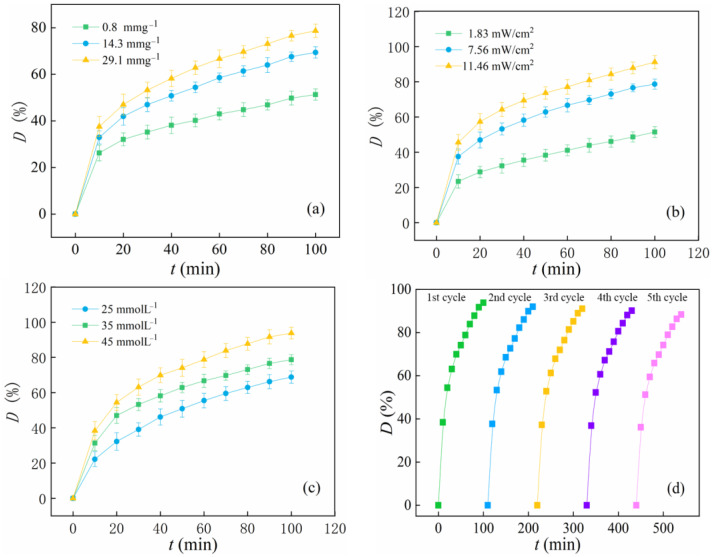
Photocatalytic performance of 0.1 g n-hemin/PAN (under H_2_O_2_/visible light) in different conditions: (**a**) hemin loading amount, (**b**) irradiation intensity, (**c**) H_2_O_2_ concentration, (**d**) recycling ability.

The catalytic property of n-hemin/PAN under visible light irradiation and H_2_O_2_ was identified by varying the catalytic conditions. Unless the catalytic condition was mentioned, the hemin loading amount was 29.1 mg g^−1^, the light intensity was 7.56 mW/cm^2^, and the concentration of H_2_O_2_ was 25 mmol L^−1^. Figure 11a illustrates the effect of the hemin loading amount on the catalytic performance of n-hemin/PAN, and the result indicates that as the hemin loading capacity grows, the n-hemin/PAN exhibits a higher decoloration rate. Especially when the hemin amount raises from 0.8 to 14.3 mg g^−1^, the RhB decoloration rate rises significantly from 51.3% to 69.41% in 100 min, and the loading amount was also used as a standard in the following discussion. It is worth mentioning that in a previous study [45], the mass of the PAN-based catalyst was about 6.7 times heavier, hence, it is clear to see that the nano-scaled carrier may support more active sites for hemin, and the synergistic interaction between hemin and PAN which has been discussed may occur. Figure 11b discusses the interaction between irradiation intensity and catalytic ability. It can be seen that there was an obvious positive correlation between the decoloration capacity of n-hemin/PAN and the light irradiation intensity, which probably means that the increase of light intensity favors the π electron transition in n-hemin/PAN. Meanwhile, the π-π stacking effect between hemin and PAN was conducive to the transfer of photogenerated electrons, which further promotes the enhancement of hemin activity. On the other hand, dyes adsorbed on fibers could also be activated by light to generate electrons and thus transfer them to the surface of hemin, and, as a result, valence variation between Fe(III) and Fe(II) in hemin could be accelerated to produce more oxidative radicals. Figure 11c shows the RhB catalytic decoloration in the varieties of H_2_O_2_ concentration where the original RhB solution was 0.02 mmol L^−1^. As the rise of H_2_O_2_ concentration occurred, especially when it reached 45 mmol L^−1^, the RhB dyes could be totally decolorized in 100 min, suggesting that the increase in H_2_O_2_ concentration contributes to the improvement of catalytic performance. The more H_2_O_2_ molecules exist in the catalytic system, the more •OH radicals are generated to oxidize the pollutants. Reusability and operational stability were vital performances for catalysts; therefore, a recycling test for n-hemin/PAN membranes was conducted, and the result is shown in Figure 11d. In the catalytic cycles, n-hemin/PAN could be easily recycled owing to the fiber membrane’s support. After five consecutive processes, n-hemin/PAN still maintained a high catalytic activity without significant decrease, it was also confirmed by the atomic absorption spectrum result in which barely any Fe(III) ions were detected. The catalytic properties of n-hemin/PAN in the study were compared with several pieces of literature [45,46], in which hemin and RhB were simultaneously conducted in the catalytic system. It was concluded that n-hemin/PAN showed higher catalytic performance per mass. Moreover, given that n-hemin/PAN was prepared by a simple one-step method with relatively low cost, n-hemin/PAN showed superiority in contaminants elimination.

**Figure 12:**
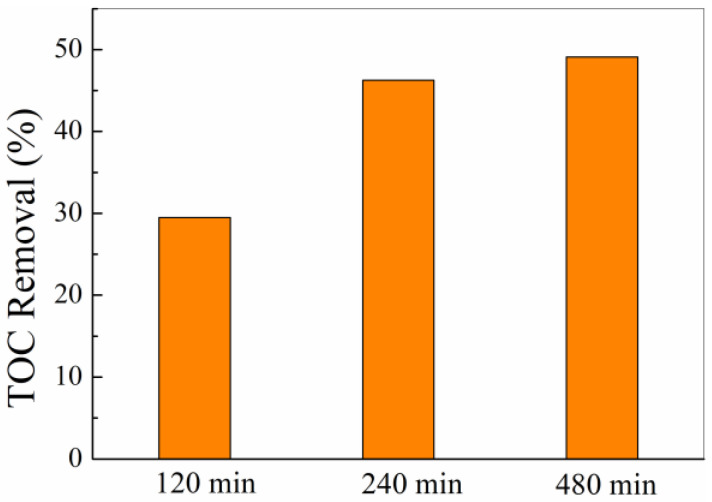
Time evolution of TOC during the RhB decoloration under visible light irradiation with H_2_O_2_.

TOC removal analysis was employed during the RhB degradation process. As is shown in Figure 12, the removal efficiency of TOC was 29.47% after a 120 min catalytic process when the dye solution was totally decolorized. At the time, the chromophoric groups in RhB dyes were destructed. However, smaller organic molecules were supposed to be generated. Hence, TOC was sequentially tested at 240 min and 480 min, and the result showed that TOC removal efficiency increased up to 46.28% and 49.10%, respectively, indicating the n-hemin/PAN was an effective catalyst to decolorize dyes and reduce TOC.

**Figure 13:**
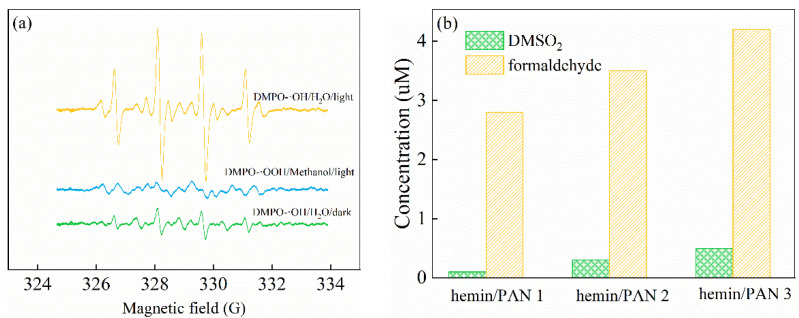
The ESR signals of DMPO-•OH and DMPO-•OOH adducts during the catalytic system (**a**), and DMSO_2_ and formaldehyde concentration (**b**) in the system of n-hemin/PAN/H_2_O_2_.

The catalytic mechanisms for hemin catalysts were divided into two types: heterolytic cleavage and homolytic cleavage of the Fe-OOH complexes. To further discuss the mechanism of the efficient catalytic system, n-hemin/PAN under H_2_O_2_ addition, an ESR technique was used in the first place to detect the oxidative radicals derived from the system. As is shown in Figure 13a, the characteristic signal with a peak intensity ratio of 1:2:2:1 was found within the n-hemin/PAN/H_2_O_2_ catalytic system, which belonged to the DMPO-•OH signals. On the other hand, characteristic intensity ascribing to the DMPO-•OOH was obviously lower than DMPO-•OH, suggesting that there was barely any amount of •OOH radicals interacted in the n-hemin/PAN catalytic system, and the decoloration effect on RhB was mostly contributed by the •OH radicals. Moreover, the DMPO-•OH intensity comparison between the dark and visible light irradiation state in the n-hemin/PAN/H_2_O_2_ system showed that •OH radicals were primarily produced under an irradiation condition, manifesting that n-hemin/PAN was a kind of light-driven catalyst, and the result also agreed with the previous discussion in Figure 11b. According to studies [10,44], the active radicals provided by the hemin molecules were mostly •OH radicals and Fe(IV)=O. Accordingly, a DMSO catalytic oxidation experiment was carried out to identify the Fe(IV)=O species in the n-hemin/PAN/H_2_O_2_ system by quantifying the oxidized products DMSO_2_ and formaldehyde. As the mechanism elucidated in the previous study [40], the Fe(IV)=O could react with the DMSO to produce DMSO_2_, while the •OH radicals could react with the DMSO to produce formaldehyde. Therefore, the oxidized products of DMSO would be distinguishable if an Fe(IV)=O species existed. The result is displayed in Figure 13b. The n-hemin/PAN/H_2_O_2_ system detected a large amount of formaldehyde and a small amount of DMSO_2_, suggesting that the dominant radicals operated in the system were mainly •OH radicals. Thus, a possible photocatalytic reaction during the process was proposed as follows:

Fe(III)-hemin + H_2_O_2_ → Fe(II)-hemin + •OOH + H^+^ (2)

Fe(III)-hemin + •OOH → Fe(II)-hemin + O_2_ + H^+^ (3)

Fe(II)-hemin + H_2_O_2_ → Fe(III)-hemin + •OH + OH^-^ (4)

Organic pollutants + •OH → degraded products(5)

## 4. Conclusions

In summary, the n-hemin/PAN nanocomposite membranes were successfully prepared utilizing hemin as the enzyme-like catalyst and PAN as a fiber support which could also improve their photo-response ability by molecular interaction. SEM, FTIR, XPS, and DRS were conducted to characterize the prepared samples in detail. In addition, n-hemin/PAN showed excellent decoloration performance when the hemin loading amount was 29.1 mg g^−1^, light irradiation intensity was 11.46 mW/cm^2^, and the H_2_O_2_ dosage was 45 mmol L^−1^. Moreover, n-hemin/PAN membranes maintained high catalytic activity after five consecutive processes. The reactivity of n-hemin/PAN was greatly affected by light irradiation, suggesting the nanocomposite membrane was a promising photo-enhanced catalyst in contaminant decomposition.

## Figures and Tables

**Table 1:** Results from linear regression of pseudo-second-order equation plots of n-hemin/PAN.

Sequence	Adsorption Condition	Rate Equation	k	q_e_	R^2^
a	Dark	t/C_t_ = 1.55 + t/4.44	0.011	4.44	0.9949
b	Visible irradiation	t/C_t_ = 2.92 + t/4.23	0. 036	4.23	0.9949
c	Dark/H_2_O_2_	t/C_t_ = 1.80 + t/4.93	0.014	4.93	0.9923
d	Visible irradiation/H_2_O_2_	t/C_t_ = 4.44 + t/8.61	0.008	8.61	0.9731

## Data Availability

Not applicable.
